# Supplementary figures and images for: Association of serum Klotho with tinnitus prevalence, duration and severity: A cross-sectional study in middle-aged and older adults
Source: PLoS One. 2025 Jul 30;20(7):e0327228. doi: 10.1371/journal.pone.0327228 (PMC12309988; doi:10.1371/journal.pone.0327228)

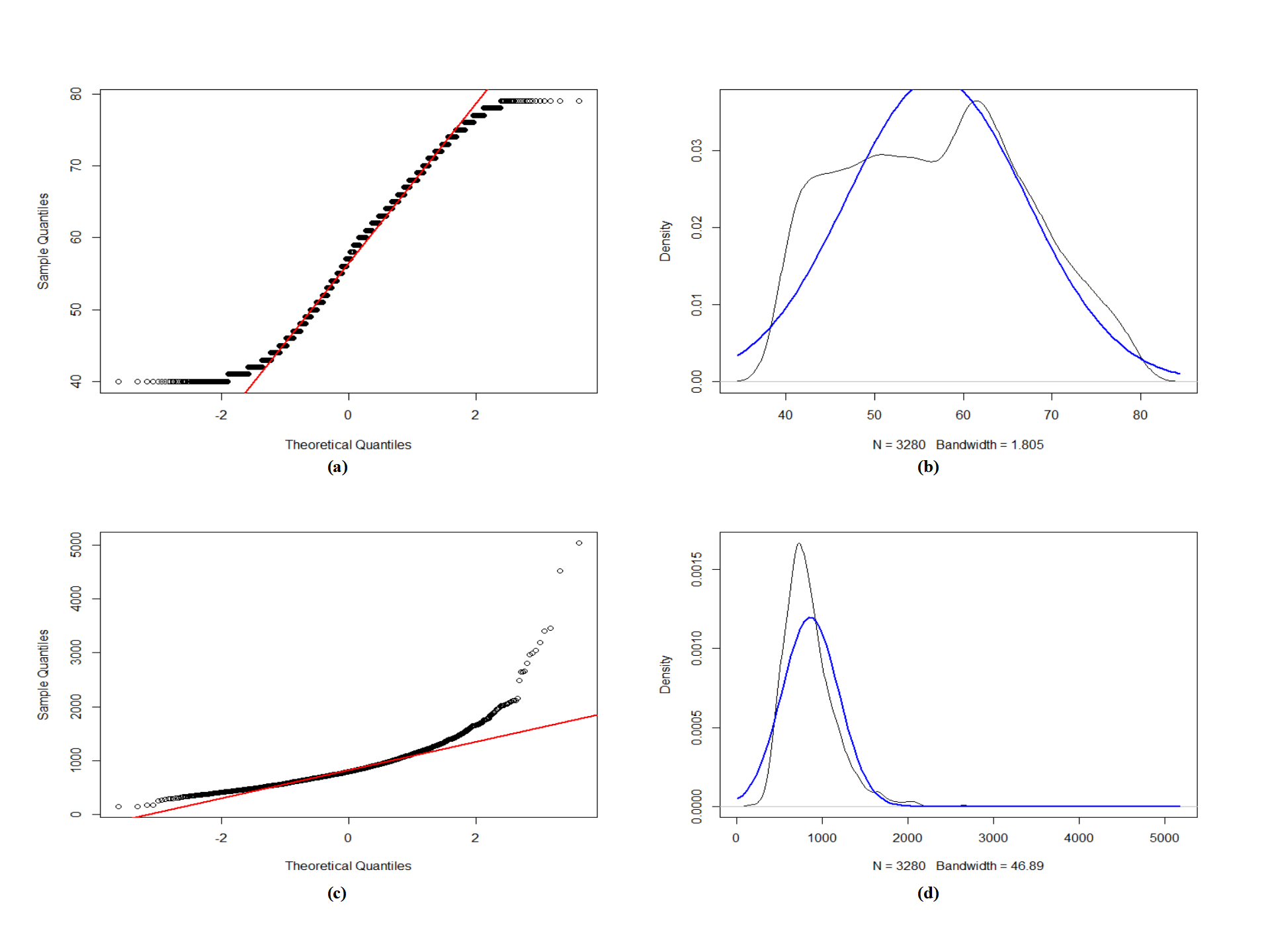

Supplement: S1 Fig — (a) Q-Q plot and (b) density plot for age; (c) Q-Q plot and (d) density plot for serum Klotho. (TIF) [file pone.0327228.s001.tif]
