# Supplementary material for: Association of serum Klotho with tinnitus prevalence, duration and severity: A cross-sectional study in middle-aged and older adults
Source: PLoS One. 2025 Jul 30;20(7):e0327228. doi: 10.1371/journal.pone.0327228 (PMC12309988; doi:10.1371/journal.pone.0327228)
Supplement: S1 Table — The classification of serum Klotho was based on the unweighted lower quartile and median of concentrations (Low: ≤ 648.75pg/mL; Medium: 648.76 ~ 779.8pg/mL; High: > 779.8pg/mL). All estimates were weighted to be nationally representative. Abbreviations: OR: odds ratio; CI: confidence interval; BMI: body mass index; PIR: poverty income ratio; PHQ-9: Patient Health Questionnaire-9; PTA: Pure-tone average. (DOC) [file pone.0327228.s002.doc]

**S1 Table.** Associations between serum Klotho and tinnitus severity.

| **Characteristic** | **Tinnitus severity** | | | | **p-value** |
| --- | --- | --- | --- | --- | --- |
| **Overall  (n=691)** | **None  (n=152)** | **Mild  (n=276)** | **Moderate–Severe (n=263)** |
| **Serum Klotho (pg/mL)** | 765 (638, 951) | 704 (585, 924) | 768 (646, 962) | 786 (638, 966) | 0.2 |
| **Serum Klotho [(%)](../../../../C:%5C1%E5%8D%9A%E5%A3%AB%5CNhanes%E6%95%B0%E6%8D%AE%E5%BA%93%5C%E8%A1%80%E6%B8%85klotho%E4%B8%8E%E8%80%B3%E9%B8%A3%5C%E6%8A%95%E7%A8%BF%5C%E6%8A%95%E7%A8%BF%5Ctable1.doc#SSKLOTH)** |  |  |  |  | 0.5 |
| **Low** | 203 (28%) | 51 (33%) | 76 (26%) | 76 (26%) |  |
| **Medium** | 186 (31%) | 37 (33%) | 75 (30%) | 74 (30%) |  |
| **High** | 302 (42%) | 64 (34%) | 125 (44%) | 113 (44%) |  |

Tinnitus severity was categorized into none (no problem), mild (a small problem) and moderate–severe (a moderate/big/very big problem). The classification of serum klotho was based on the unweighted lower quartile and median of concentrations (Low: ≤648.75pg/mL; Medium: 648.76~779.8pg/mL; High: >779.8pg/mL). All estimates were weighted to be nationally representative.
