# Supplementary material for: Association of serum Klotho with tinnitus prevalence, duration and severity: A cross-sectional study in middle-aged and older adults
Source: PLoS One. 2025 Jul 30;20(7):e0327228. doi: 10.1371/journal.pone.0327228 (PMC12309988; doi:10.1371/journal.pone.0327228)
Supplement: S2 Table — Tinnitus severity was categorized into none (no Problem), mild (small problem) and moderate–severe (moderate/big/very big problem). The classification of serum Klotho was based on the unweighted lower quartile and median of concentrations (Low: ≤ 648.75pg/mL; Medium: 648.76 ~ 779.8pg/mL; High: > 779.8pg/mL). All estimates were weighted to be nationally representative. (DOC) [file pone.0327228.s003.doc]

**S2 Table.** Univariate logistic regression of serum Klotho and tinnitus prevalence.

| **Characteristic** | **OR (95% CI)** | **P-value** |
| --- | --- | --- |
| **Age** (years) |  |  |
| <60 | 1 (reference) |  |
| ≥60 | 1.432 (1.210, 1.695) | <0.001 |
| **Gender** |  |  |
| Female | 1 (reference) |  |
| Male | 1.144 (0.967, 1.353) | 0.116 |
| **Race** |  |  |
| Non-Hispanic White | 1 (reference) |  |
| Non-Hispanic Black | 0.657 (0.528, 0.814) | <0.001 |
| Mexican American | 0.948 (0.747, 1.197) | 0.656 |
| Other Hispanic | 0.712 (0.539, 0.931) | 0.015 |
| **BMI** (kg/m2) |  |  |
| <25 | 1 (reference) |  |
| 25-29.9 | 0.963 (0.753, 1.235) | 0.765 |
| ≥30 | 1.291 (1.030, 1.628) | 0.028 |
| **Educational level** |  |  |
| Below high school | 1 (reference) |  |
| Completed high school | 0.930 (0.734, 1.179) | 0.55 |
| Beyond high school | 0.817 (0.667, 1.003) | 0.052 |
| **Marital status** |  |  |
| Married or living with partner | 1 (reference) |  |
| Single, divorced or widowed | 1.254 (1.056, 1.488) | 0.01 |
| **PIR** |  |  |
| ≤1 | 1 (reference) |  |
| >1 | 0.756 (0.619, 0.927) | 0.007 |
| **Alcohol status** |  |  |
| No | 1 (reference) |  |
| Yes | 1.000 (0.828, 1.211) | 0.998 |
| **Smoking status** |  |  |
| No | 1 (reference) |  |
| Yes | 1.437 (1.214, 1.702) | <0.001 |
| **Diabetes** |  |  |
| No | 1 (reference) |  |
| Borderline | 1.248 (0.766, 1.963) | 0.354 |
| Yes | 1.373 (1.115, 1.685) | 0.003 |
| **Hypertension** |  |  |
| No | 1 (reference) |  |
| Yes | 1.620 (1.368, 1.920) | <0.001 |
| **Total cholesterol** [(mg/dL)](https://wwwn.cdc.gov/Nchs/Data/Nhanes/Public/2015/DataFiles/TCHOL_I.htm#LBXTC) |  |  |
| <240 | 1 (reference) |  |
| ≥240 | 0.950 (0.751, 1.193) | 0.665 |
| **Cardiovascular disease** |  |  |
| No | 1 (reference) |  |
| Yes | 1.970 (1.567, 2.468) | <0.001 |
| **PHQ-9** |  |  |
| <10 | 1 (reference) |  |
| ≥10 | 2.607 (2.048, 3.309) | <0.001 |
| **Noise exposure** |  |  |
| No | 1 (reference) |  |
| Yes | 1.875 (1.584, 2.221) | <0.001 |
| **PTA** (dB) |  |  |
| <25 | 1 (reference) |  |
| ≥25 | 2.721 (2.290, 3.234) | <0.001 |
| **ln Klotho**（pg/mL) | 0.690 (0.540, 0.880) | 0.003 |
| **Serum Klotho** |  |  |
| Low | 1 (reference) |  |
| Medium | 0.886 (0.705, 1.112) | 0.297 |
| High | 0.684 (0.560, 0.838) | <0.001 |

ln(Klotho): natural log-transformed serum Klotho concentrations. The classification of serum klotho was based on the unweighted lower quartile and median of concentrations (Low: ≤648.75pg/mL; Medium: 648.76~779.8pg/mL; High: >779.8pg/mL). All estimates were weighted to be nationally representative. Abbreviations: OR, odds ratio; 95% CI, 95% confidence interval; BMI: body mass index; PIR: poverty income ratio; PHQ-9: Patient Health Questionnaire-9; PTA: Pure-tone average.
