# Supplementary material for: Association of serum Klotho with tinnitus prevalence, duration and severity: A cross-sectional study in middle-aged and older adults
Source: PLoS One. 2025 Jul 30;20(7):e0327228. doi: 10.1371/journal.pone.0327228 (PMC12309988; doi:10.1371/journal.pone.0327228)
Supplement: Attachment — Submitted filename: Response to Reviewers.doc [file pone.0327228.s005.doc]

Subject: Response to Reviewer Comments for Manuscript PONE-D-25-25271

Dear Dr. Caruso,

I hope this message finds you well. I am writing in response to the comments and suggestions provided by the reviewers for our manuscript entitled "Association of serum Klotho with tinnitus prevalence, duration and severity: A cross-sectional study in middle-aged and older adults" submitted to PLOS ONE.

We greatly appreciate the time and effort you and the reviewers spent to evaluate our manuscript. We found their comments to be constructive and insightful, which significantly contributed to the improvement of our manuscript. We have carefully addressed each point and made necessary changes to the manuscript accordingly. Please find our detailed responses to each journal requirements and comments below.

**Journal requirements:**

Requirement 1:

[Please ensure that your manuscript meets PLOS ONE's style requirements]

Response 1:

We have carefully revised the manuscript to fully comply with PLOS ONE's style requirements.

Requirement 2:

[Please state what role the funder took in the study.]

Response 2:

We sincerely appreciate your valuable comments on our manuscript. As requested, we have confirmed the following statement regarding the role of the funder in the revised manuscript:
"This work was supported by the National Natural Science Foundation of China (Grant No. 82160213). The funder had no role in study design, data collection and analysis, decision to publish, or preparation of the manuscript."

Requirement 3:

[Data availability statement.]

Response 3:

Thank you for your feedback. We confirmed that this study utilizes publicly available data from the NHANES database, which is freely accessible online. We have updated the Data Availability Statement in the manuscript as follows: "The data supporting this study are publicly available from the NHANES database at <https://www.cdc.gov/nchs/nhanes/>." Since no original data were generated in this study (only secondary analysis of NHANES data), no additional data deposition is required. Please let us know if further clarification is needed.

Requirement 4:

[There is identifying data in the Supporting Information file “S1 File.csv”.]

Response 4:

Thank you for your note regarding the supporting file. We have carefully reviewed PLOS ONE's Data Policy and confirm: All data in this study are derived from the publicly available, de-identified NHANES database. No original participant-level data were collected. This file contained intermediate summary statistics generated from NHANES data, which are fully reproducible using the NHANES raw data (access instructions provided in Methods); Therefore, we decided to remove S1 File.csv from the submission to avoid any potential misinterpretation of data sensitivity.

**Reviewer 1:**

Comment 1:

[In table 1 the term “Race” should be avoided and substituted with “Ethnic origin”.]

Response 1:

We thank the reviewer for raising this important point. we have substituted "Race" with “Ethnic origin” throughout the entire manuscript based on reviewer’s proposal.

Comment 2:

[Given age as a potential confounder, It is possible that analyzing data for age cohorts a better clear cut evidenced results might be obtained for the evaluation of klotho tinnitus relationship. In addition, data on klotho levels reported in table 1 might be biased by age.]

Response 2:

We appreciate reviewer's insightful observation and suggestion for this issue. The influence of age on the Klotho-tinnitus relationship should indeed be seriously considered. To address this, we have further assessed the Klotho-tinnitus relationship through age-stratified regression analyses with four age group including 40-49, 50-59, 60-69 and 70-79 (Table 4). Meanwhile, corresponding supplements were also made in the sections of Abstract, Methods (lines 183-185), Results (lines 257-285), Discussion (lines 320-338) and Conclusion (lines 405-408). The supplementary or modified content were marked in red font and underline for visibility.

Comment 3:

[Maybe the analyses should be performed on positive only to clearly establishing the relationship between reduction of klotho levels and tinnitus.]

Response 3:

We sincerely appreciate the reviewer’s insightful suggestion. We fully agree with the reviewer that analyzing the tinnitus-positive subgroup could further clarify the relationship between serum Klotho levels and tinnitus characteristics (e.g., duration or severity). As presented in Table 2 and Table S1, preliminary analyses in 691 tinnitus-positive participants revealed no significant associations between Klotho and tinnitus duration or severity (all p >0.05). Consequently, multivariate logistic regression was not pursued for these outcomes, as the data did not support a meaningful association. This limitation may stem from insufficient statistical power or unmeasured confounders, which we aim to address in future studies with larger cohorts. Thank you for this constructive suggestion, which undoubtedly strengthens our study.

In addition, In Table 3, we employed multivariable logistic regression models including both tinnitus-positive and -negative subjects to examine the association between serum Klotho levels and tinnitus prevalence, as this is a standard approach for assessing risk factors for binary outcomes (presence/absence of tinnitus).

Comment 4:

[In discussion, It is suggested to add some relevant contents: (1) FGF-23-Klotho endocrine axis may play a novel role in the inner ear regulating directly or indirectly the kinocilium-stereocilia interaction; (2) Klotho reduction effect on activity and protein abundance of KCNQ1/KCNE1, a K(+) channel required for proper hearing.]

Response 4:

We have supplemented relevant contents displayed with a red font and underline for visibility based on reviewer’s proposal. Corresponding to (1) in lines 359-361 and (2) in lines 375-377 respectively.

Comment 5:

[The limitation 4“The study was conducted in a single geographical location, which may introduce biases and limit the applicability of the results to broader populations” appears not justified.]

Response 5:

We have revised relevant contents with the limitation 4 in lines 399-403.

Additionally, to provide more intuitive effect size estimates, we have added standardized mean differences (SMD) in Table 1, and described it specifically in the Statistical Analysis of Methods (lines 164-173). Furthermore, to better elucidate the relationship between klotho and tinnitus, we consolidated Model 2 and Model 3 from Table 3 into a single Model 3, thereby streamlining the analysis from four models to three (lines 236-256).

We believe that the changes made in response to the reviewers' comments have significantly improved our manuscript, and we are confident that it is now suitable for publication in PLOS ONE. Please do not hesitate to contact us should there be any additional comments or suggestions, or if you require further clarification on the changes we have made.

Thank you very much for considering our responses, and we look forward to your positive reply.

Best regards,

Shi-Hua Yin, MD

ENT & HN Surgery Department,

The Second Affiliated Hospital of Guangxi Medical University,

Da-Xue Dong Road No.166, Nanning, Guangxi, China,

Tel.: +8613978865695,

E-mail address: shihuayin@gxmu.edu.cn
